# Transcriptome-wide identification and characterization of the *Sox* gene family and microsatellites for *Corbicula fluminea*

**DOI:** 10.7717/peerj.7770

**Published:** 2019-10-22

**Authors:** Chuankun Zhu, Lei Zhang, Huaiyu Ding, Zhengjun Pan

**Affiliations:** 1 Jiangsu Engineering Laboratory for Breeding of Special Aquatic Organisms, Huaiyin Normal University, Huai’an, Jiangsu, China; 2 Jiangsu Collaborative Innovation Center of Regional Modern Agriculture & Environmental Protection, Huaiyin Normal University, Huai’an, China; 3 Key Laboratory of Fishery Sustainable Development and Water Environment Protection of Huai’an City, Huai’an Sub Center of the Institute of Hydrobiology, Chinese Academy of Sciences, Huai’an, China

**Keywords:** *Corbicula fluminea*, Transcriptome, *Sox* gene family, Microsatellite

## Abstract

The Asian clam, *Corbicula fluminea*, is a commonly consumed small freshwater bivalve in East Asia. However, available genetic information of this clam is still limited. In this study, the transcriptome of female * C. fluminea* was sequenced using the Illumina HiSeq 2500 platform. A total of 89,563 unigenes were assembled with an average length of 859 bp, and 36.7% of them were successfully annotated. Six members of *Sox* gene family namely *SoxB1*, *SoxB2*, *SoxC*, *SoxD*, *SoxE* and *SoxF* were identified. Based on these genes, the divergence time of *C. fluminea* was estimated to be around  476 million years ago. Furthermore, a total of 3,117 microsatellites were detected with a distribution density of 1:12,960 bp. Fifty of these microsatellites were randomly selected for validation, and 45 of them were successfully amplified with 31 polymorphic ones. The data obtained in this study will provide useful information for future genetic and genomic studies in *C. fluminea*.

## Introduction

The Asian clam, *Corbicula fluminea* (Corbiculidae), is native to the East and Southeast of Asia (Araujo, Moreno & Ramos, 1993), and it is common in rivers and lakes of China. *C. fluminea* is a filter-feeder, and hence, it plays an important role in the maintenance of hydroecological balance in its original habitats. However, in other areas of the world, especially Europe and North America, this clam is believed to be invasive and threatening to native aquatic communities (Gatlin, Shoup & Long, 2013; Crespo et al., 2015). Nevertheless, because this clam is nutritious and delicious, it is well-liked by East Asian consumers. In China, *C. fluminea* is an important aquaculture bivalve, and it has become a dominant export aquatic product in some areas such as the Hongze Lake. However, owing to increasing market demand and water pollution, natural resources of *C. fluminea* have sharply declined; for example, the annual production of *C. fluminea* in the Hongze Lake has decreased from 100,000 tons to 22,000 tons in recent years (Liu et al., 2018). Worse still, the economic traits have also declined (Wang & Chang, 2010), and therefore, germplasm and resource conservations for *C. fluminea* are urgently needed. Genomic and genetic studies on *C. fluminea* are limited, as most studies have focused on environmental monitoring and invasion control (Crespo et al., 2015; Falfushynska, Phan & Sokolova, 2016; Bertrand et al., 2017).

*Sry*-related high-mobility group box genes (*Sox*) are believed to be an ancient gene family, and they have been widely used as a powerful toolkit in studies on animal phylogenesis (Phochanukul & Russell, 2010), genomic evolution (Heenan, Zondag & Wilson, 2016), gene development (Wei et al., 2016), and gene duplication (Guo, Tong & He, 2009). The *Sox* gene family exists in almost all animals from the most basal lineage such as choanoflagellate to higher animals including human (Heenan, Zondag & Wilson, 2016). The first identified *Sox* gene was *Sry*; it carries a DNA-binding high mobility group (HMG) box and is associated with the mammalian testis determination (Gubbay et al., 1990). Presently, more members of the *Sox* family have been identified with more than 40 *Sox* genes being determined in animal genomes. *Sox* genes are divided into 11 groups (A-K) primarily according to the similarity of their HMG box (Wei et al., 2016; Yu et al., 2017). The number of *Sox* genes varies among different animals, and it is generally believed that vertebrates have more *Sox* genes than invertebrates. Among the 11 *Sox* groups, *SoxB*, *SoxC*, *SoxD, SoxE* and *SoxF* are believed to be core *Sox* subgroups (Heenan, Zondag & Wilson, 2016), as these genes could be found in almost all animals including the most basal animals (Fortunato et al., 2012). Transcription factors of *Sox* genes have various functions in the growth and developmental processes of animals. *Sry*, *Sox3*, *Sox5*, *Sox6*, *Sox8*, *Sox9* and *Sox17* are related to testicle development and sex determination (Gubbay et al., 1990; Graves, 1998; Frojdman, Harley & Pelliniemi, 2000; Furumatsu & Asahara, 2010); *Sox1*, *Sox2* and *Sox3* are related to neurogenesis (Hong & Saint-Jeannet, 2005); and *Sox7*, *Sox8*, *Sox9*, *Sox10* and *Sox18* are associated with vascular development and arteriovenous specification (Montero et al., 2002; Cermenati et al., 2008; Herpers et al., 2008). Although, Mollusca represents the second largest animal group, studies on their *Sox* genes are quite limited; there are only a few reports on limpet (Le Gouar, Guillou & Vervoort, 2004), scallop (He et al., 2013; Yu et al., 2017), abalone (O’Brien & Degnan, 2000), oyster (Zhang, Xu & Guo, 2014b), and cephalopod (Focareta & Cole, 2016). As no study on *Sox* genes of *C. fluminea* has been reported, the *Sox* family in this species is presently unknown.

Molecular markers are useful tools for resource protection and economic trait improvement in aquatic animals (Tong & Sun, 2015). Microsatellite (also known as simple sequence repeat, SSR) is a widely used molecular marker, and because of the advantages of wide distribution, high polymorphism, codominant inheritance, as well as high stability and repeatability, this marker is preferred by researchers (Chistiakov, Hellemans & Volckaert, 2006). In recent years, microsatellites have been used in genetic and genomic studies including population polymorphism analysis, genetic linkage map construction, quantitative trait loci (QTL) identification and marker assisted selection breeding (MAS) of many aquatic animals (Yue, 2014; Tong & Sun, 2015).

Along with the development of sequencing technology, high-throughput sequencing such as RNA sequencing (RNA-seq) has become an efficient method for obtaining genes and other genomic information of non-model organisms, as well as for the isolation of molecular markers. Transcriptome assembly from RNA-seq data is an effective and efficient approach for massive functional gene identification and SSR development in mollusks and other aquatic organisms (Gao et al., 2012; Li et al., 2015a; Chen et al., 2016; Qin et al., 2012; Werner et al., 2013). To date, transcriptome information has been acquired in many mollusks for the purpose of functional gene isolation (Niu et al., 2016; Wang, Liu & Wu, 2017b), molecular marker development (Chen et al., 2016; Kang et al., 2016), sex determination (Teaniniuraitemoana et al., 2014; Li et al., 2016) and evolution analyses (Liscovitch-Brauer et al., 2017; Gorbushin, 2018).

A previous transcriptome of mixed sample of five tissues (mantle, muscle, digestive gland, gonad and gill) has been reported in *C. fluminea* using the Illumina GAIIx method, and 15 functional genes were identified as potential environmental pollution biomarkers (Chen et al., 2013). In the present study, the transcriptome of whole soft tissue was sequenced using the Illumina HiSeq 2500 platform and the unigenes were assembled, characterized and annotated for the purpose of identifying *Sox* genes and obtaining SSRs in *C. fluminea*. The data acquired in this study will supply valuable information for future genetic and genomic studies including functional gene analyses, genomic evolution, natural resource and germplasm conservation, linkage map construction, QTL identification and MAS breeding on this clam.

## Materials & Methods

### Sample preparation and Illumina sequencing

A total of 49 *C. fluminea* collected from the Hongze Lake was used in this study, and foot tissues of 46 individuals were sampled. The genomic DNA, which was used for microsatellite validation, was extracted using the phenol-chloroform extraction protocol (Sambrook & Russell, 2001). The remaining three females were cultured in a glass tank for two days. After the excretion of silt and faeces, the whole soft tissues were collected, placed in liquid nitrogen to freeze, and stored at −80 °C until use. Total RNA was extracted using the TRIzol Reagent (Invitrogen, USA) following the manufacturers’ instructions. Next, RNA was treated with DNase I (Takara, Japan) at 37 °C for 45 min to remove residual DNA, and was quantified by Nanodrop 2000 (Thermo Scientific, USA). Finally, 100 ng RNA from each of the samples from the three females were mixed together for library construction.

The mRNA with poly (A) was isolated from total RNA using Magnetic Oligo (dT) Beads (Invitrogen, USA). The fragmentation buffer was used to cut mRNA randomly, and cDNA was synthesized using these fragments as templates and purified by AMPure XP beads (Beckman, USA). This was followed by end repair, adenine addition, and Illumina adapter ligation of purified cDNA. Using AMPure XP beads, fragments with suitable lengths were selected and used as templates for PCR amplification. Finally, the library was sequenced using high-throughput approach by the Illumina HiSeq 2500 platform (Biomarker Technologies Co., Ltd., Beijing, China) following the manufacturer’s instructions (Illumina, San Diego, CA, USA).

### De novo assembly and unigene annotation

The softwares of SeqPrep (https://github.com/jstjohn/SeqPrep) and Condetri_v2.0.pl (http://code.google.com/p/condetri/downloads/detail?name=condetri_v2.0.pl) were used to trim raw data by discarding dirty reads including highly redundant sequences, adaptors, reads with high frequency of ambiguous bases (>10%), and low quality reads (*Q*-value < 30). Next, the Trinity software (Grabherr et al., 2011) was utilized to carry out *de novo* assembly for these trimmed high-quality clean reads.

Annotations for all assembled unigenes were implemented through BLAST search against public databases including non-redundant protein database (nr, NCBI), Gene Ontology (GO), Protein family (Pfam), Swiss-Prot, Clusters of Orthologous Groups (COG), Eukaryotic Ortholog Groups (KOG), and Kyoto Encyclopedia of Genes and Genomes (KEGG) with an *E*-value cut off of 10^−5^. The program Blast2GO (Conesa et al., 2005) was used to predict GO terms for unigenes, and the software WEGO (Ye et al., 2006) was used to classify GO functions and analyze the overall function distribution of genes for *C. fluminea*. Sequences without significant hits in the above databases were searched against the Rfam database (release 14.1; Kalvari et al., 2017) to analyze their homology with noncoding RNAs (ncRNA).

### *Sox* gene identification and characterization

According to a present study on *Sox* gene family of *Mizuhopecten yessoensis* (Yu et al., 2017), sequences of seven *Sox* genes were downloaded from GenBank (KY523526 –KY523532). The SMART software (Letunic, Doerks & Bork, 2012) was used to identify and retrieve amino acid sequences of HMG domains for these genes. HMG sequences were then used as queries to search homologous unigenes through local tBLASTn in *C. fluminea* transcriptome data with an *E*-value threshold of 10^−5^. A reciprocal tBLASTn was also carried out to confirm the identity of *C. fluminea Sox* genes, and they were named according to their homologies with the highest identification rates and lowest E-values. ClustalX 1.8 was used to compare HMG domains of identified SOX, and conserved motifs were shown using the online software Sequence Manipulation Suite (http://www.bio-soft.net/sms/).

In order to perform phylogenetic analysis of *C. fluminea Sox* genes and determine their groups, SOX proteins of human (*Homo sapiens*), zebrafish (*Danio rerio*), Yesso scallop (*Mizuhopecten yessoensis*), Pacific oyster (*Crassostrea gigas*), octopus (*Octopus bimaculoides*), and sea urchin (*Strongylocentrotus purpuratus*) were downloaded from NCBI, and their HMG domains were retrieved using SMART. Multiple alignments for HMG amino acid domains of these SOX proteins were performed using the software ClustalX 1.83 with default settings. Furthermore, minimum-evolution (ME) phylogenetic tree of the SOX proteins was constructed with the MEGA 4.0 program using human TCF7 (NM_201632) as the outgroup under the Dayhoff Matrix Model with a bootstrap replicate of 1,000. Using concatenated dataset of *SoxB1*, *SoxB2*, and *SoxD* from *C. fluminea*, *M. yessoensis*, *C. gigas*, *O. bimaculoides*, and *S. purpuratus*, another linearized tree was constructed to estimate the emergence time of *C. fluminea* through the UPGMA method under the modified Nei-Gojobori (p-distance) model (Zhong, Yu & Tong, 2006), with a transition/transversion ratio of 2 and 1,000 bootstrap replicates. All accession IDs of *Sox* genes that were used for phylogenetic analysis are listed in Table S1.

### Microsatellite isolation and validation

Unigenes with a length of more than 1,000 bp were used for microsatellite detection through the program MISA (https://webblast.ipk-gatersleben.de/misa/). Minimum repeat times for core motifs were set to ten for mono-nucleotide, six for di- nucleotides, and five for tri-, tetra-, penta- and hexa- nucleotides, respectively. For the microsatellites with enough flanking sequence lengths, primers were designed using the online software Primer 3 (Rozen & Skaletsky, 2000) under the following parameter settings: primer lengths were from 20 to 25 bases (22 bases was optimum) with a product size of 100–250 bp; annealing temperature was optimum at 50 °C to 60 °C; and the values of other parameters were at the default settings.

Fifty SSRs with multiple nucleotide repeats were randomly selected for validation. The polymorphism of the SSRs was tested in ten *C. fluminea* samples, and the characterization of polymorphic SSRs was analyzed in a test population with 36 individuals. PCR was performed in a total volume of 12.5 µL, including 50 ng of template DNA, 1.3 µL of 10 × reaction buffer, 0.4 µL of dNTP (2.5 mmol/L), 0.4 µL of forward and reverse primer mix (2.5 µmol/L), 1 U of *Taq* polymerase (CWBIO, China), and 9.4 µL sterile water. A 96-well thermal cycler (T100, BioRad) was used to perform PCRs at the following conditions: an initial denaturation at 94 °C for 4 min, followed by 35 cycles of denaturation at 94 °C for 40 s, annealing at optimal temperature for 40 s, extension at 72 °C for 45 s, and a final extension at 72 °C for 7 min. PCR products were genotyped through electrophoresis in 8% non-denaturing polyacrylamide gels and visualized through silver staining. Allele size for each locus was estimated by referring to the *pBR322/MspI* DNA marker (TianGen, China) and the Super DNA Marker (CWBIO, Beijing, China). For data analyses, the Arlequin version 3.01 software (Schneider, Roessli & Excoffier, 2000) was used to calculate the number of alleles (*Na*), observed (*Ho*) and expected (*He*) heterozygosity. In addition, MS-TOOLS (Park, 2001) was used to analyze polymorphism information content (*PIC*) for each locus.

## Results

### Illumina sequencing and de novo assembly

A total of 23,972,287 clean reads containing 5,993,071,750 clean nucleotides were generated after quality filtration of raw data, and all of these reads have been submitted to the Sequence Read Archive database of NCBI (SRA accession IDs: SRX2786025 and SRR5512046). The average GC content of the clean reads was 43.03%, and the proportion of nucleotides with quality value higher than 30 in reads (Q30) was 94.94%. After assembling clean reads using the Trinity program, 114,271 transcripts (109,298,083 nucleotides in total) were obtained with an average length of 957 bp and an N50 length of 1,299 bp (Table 1). All transcripts were more than 300 bp in length, 62.3% of which were longer than 500 bp. The transcripts were further clustered and assembled into 89,563 unigenes (Supplemental Information 2). All unigenes were longer than 300 bp with average and N50 lengths of 859 bp and 1072 bp, respectively (Table 1). Of the 89,563 unigenes, 58.8% (52,693) were longer than 500 bp, and 23.3% (20,846) were longer than 1 kb (Table 1).

**Table 1:** Statistical summary of the *de novo* transcriptome assembly for *Corbicula fluminea*.

Length range	Transcript	Unigene
300–500	43,097	36,870
500–1,000	39,299	31,847
1,000–2,000	20,539	14,181
2,000 +	11,336	6,665
Total number	114,271	89,563
Total length	109,298,083	76,960,817
N50 length	1,299	1,072
Mean length	957	859

**Table 2:** Summary of functional annotations for unigenes of *Corbicula fluminea*.

Annotated Database	Annotated unigenes	300 <= length < 1,000	length >= 1,000
COG	7,654	2,663	4,991
GO	10,783	5,135	5,648
KEGG	8,139	3,118	5,021
KOG	18,094	7,822	10,272
Pfam	21,015	8,566	12,449
Swissprot	18,593	7,599	10,994
Nr	32,178	16,495	15,683
All Annotated	32,912	17,090	15,822

**Figure 1:**
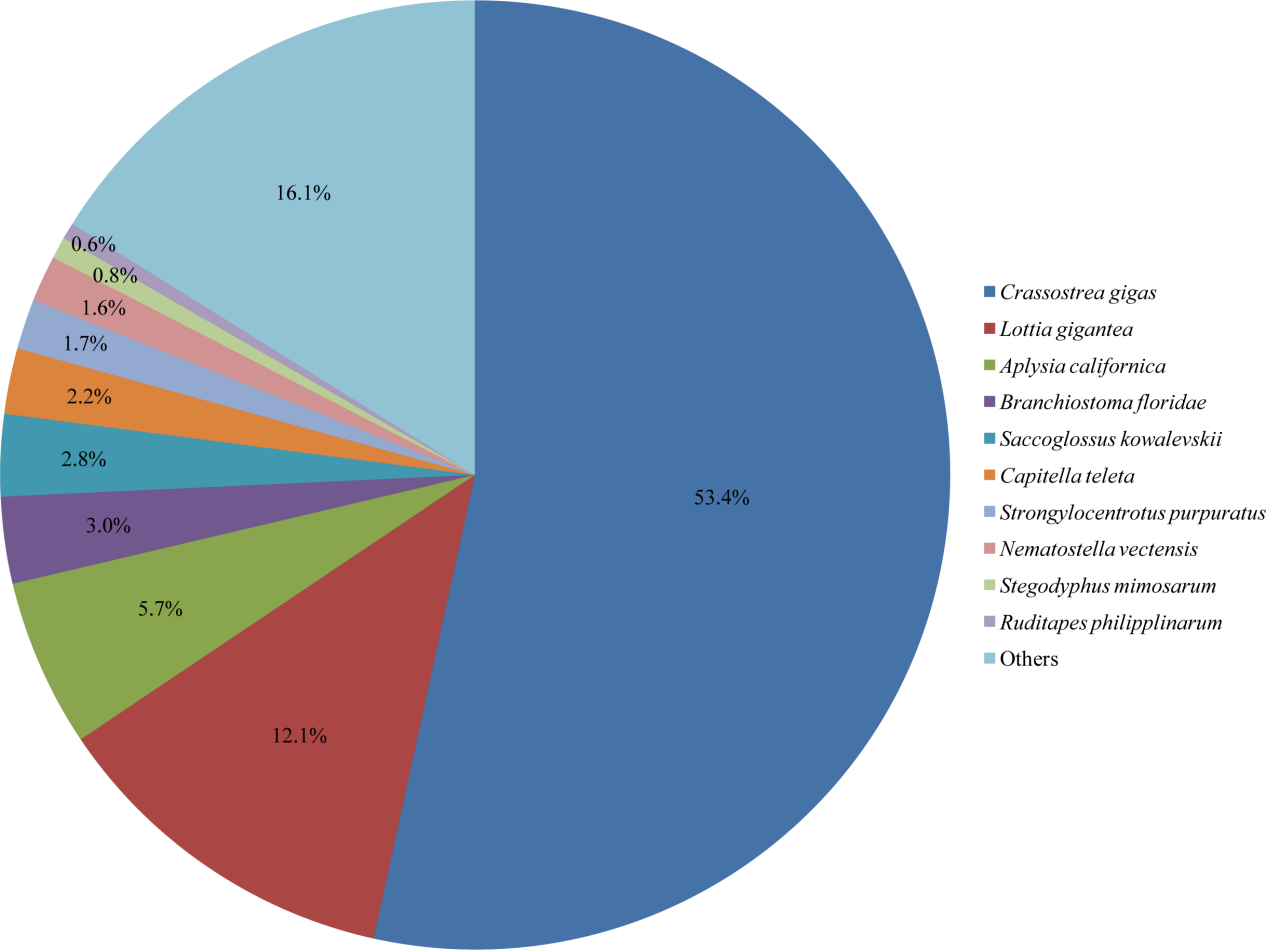
Species distribution of *Corbicula fluminea* homologies against the nr database.

### Functional annotation of unigenes

The results of functional annotation showed that 32,912 (36.7%) of the 89,563 unigenes were annotated against databases of nr, COG, GO, KEGG, KOG, Pfam, and Swissprot, among which nr contained the most homologies (Table 2, Table S2). In the nr database, the annotation rates for unigenes were the highest in *Crassostrea gigas* (53.4%), followed by *Lottia gigantea* (12.1%) (Fig. 1). The annotated sequences for unigenes were all longer than 300 bp, 15,822 (48.1%) of which were longer than 1 kb (Table 2). Additionally, the rest 56,651 (63.3%) unigenes, which had no BLAST hits in these databases, were further searched in the Rfam database and the results showed that 256 (0.5%) of them were homologous with ncRNAs, including 141 (55.1%) rRNA, 83 (32.4%) tRNA, and 32 (12.5%) other types (Table S3).

**Figure 2:**
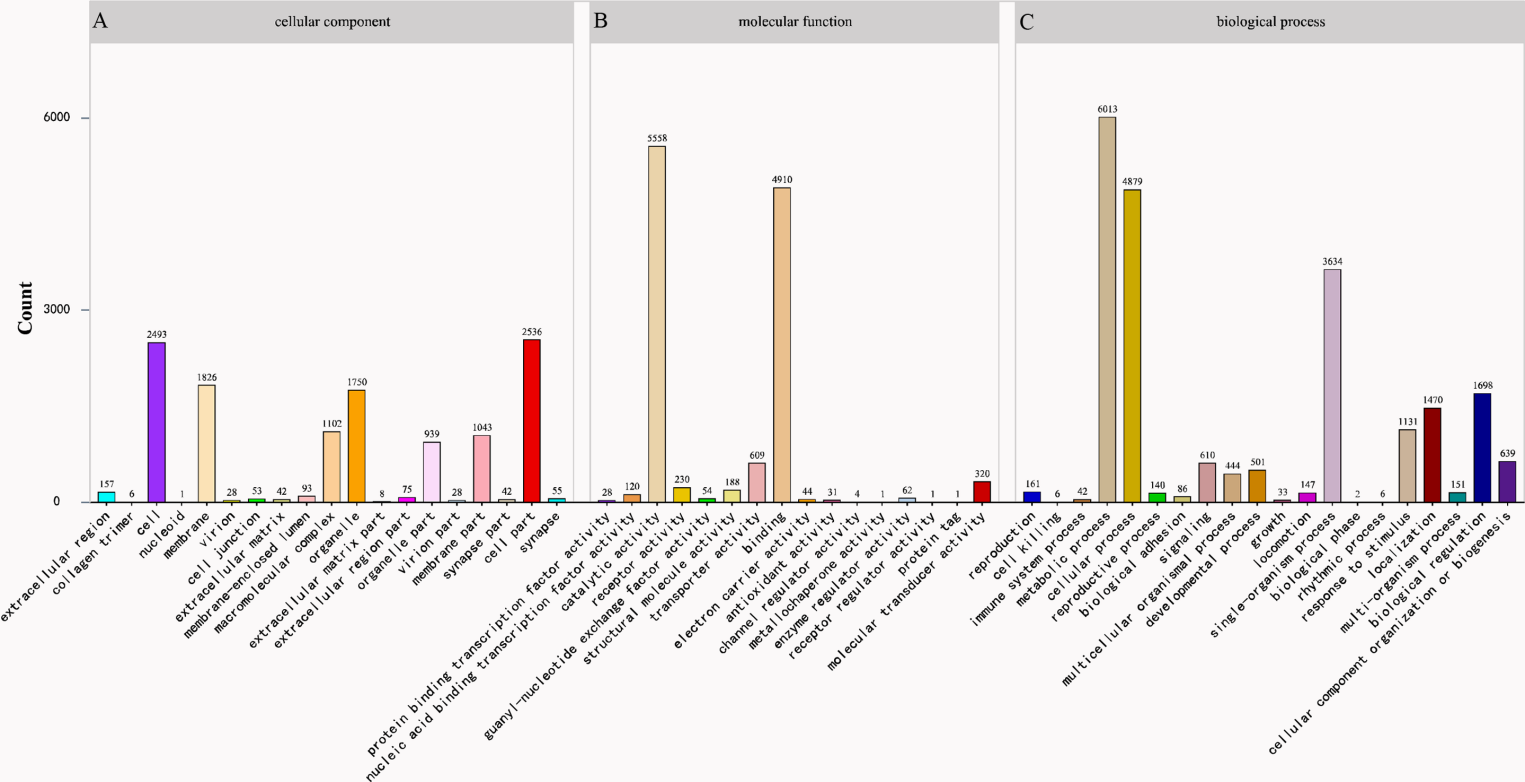
Gene Ontology (GO) classification of * Corbicula fluminea* assembled unigenes. (A) Cellular component, (B) molecular function, (C) biological process.

The program Blast2GO was utilized for the classification of the predicted functions of unigenes into three categories: cellular component, molecular function, and biological process. The category “biological process” consisting of 20 functional groups showed the highest number of annotations with metabolic process being the dominant group (27.6%), followed by cellular process (22.4%) (Fig. 2). The “cellular component” category consisted of 19 functional groups with most unigenes related to terms of cell part (20.7%) and cell (20.3%) (Fig. 2). For the category of “molecular function”, 16 functional groups were predicted with catalytic activity (45.7%) and binding (40.4%) being dominant terms (Fig. 2).

**Figure 3:**
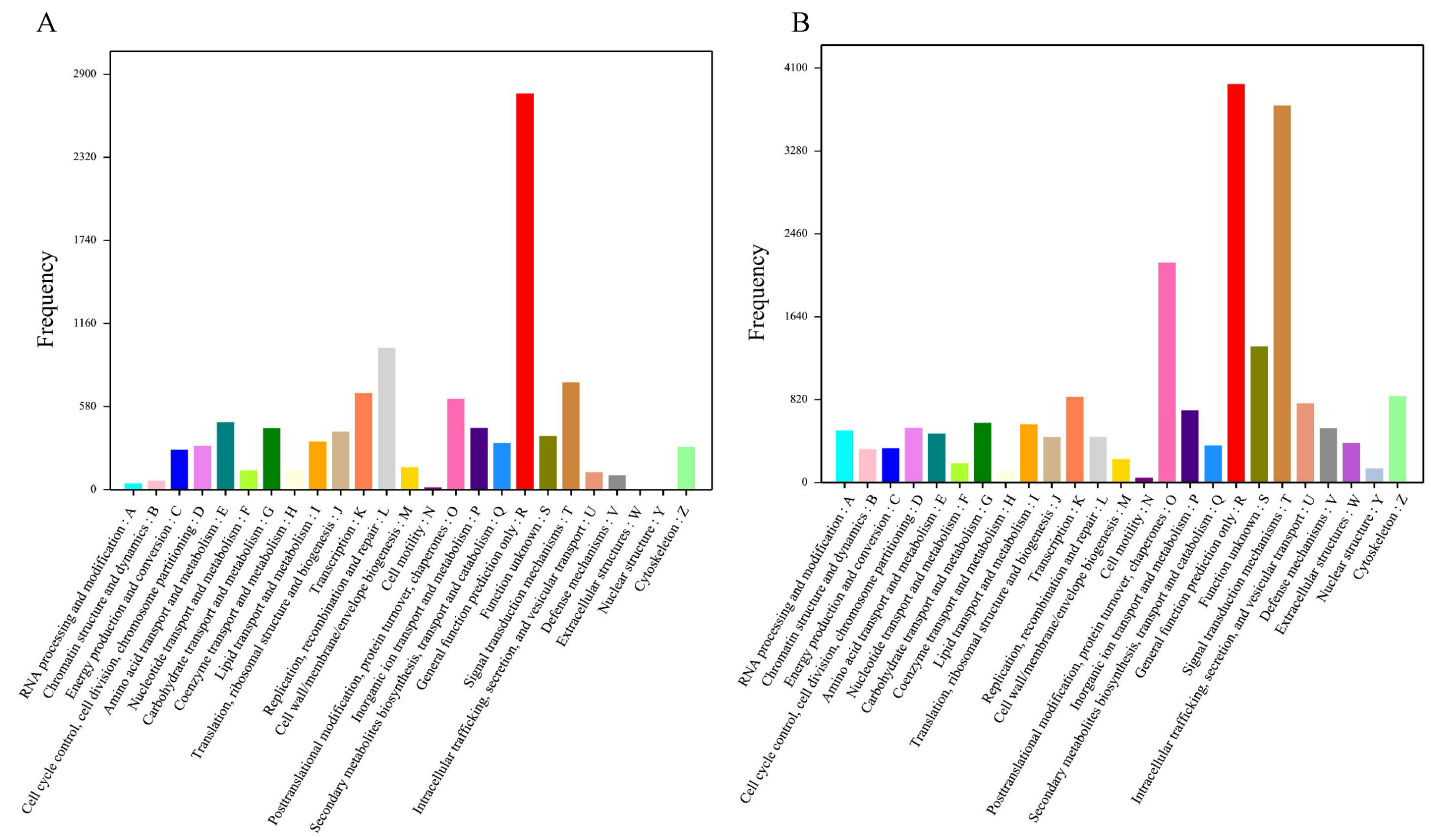
Functional classification of *Corbicula fluminea* unigenes. (A) COG (Clusters of Orthologous Groups) functional classification of unigenes, (B) KOG (Eukaryotic Ortholog Groups) functional classification of unigenes. A–Z stand for 25 COG and KOG functional classifications.

A total of 7,654 unigenes were annotated in the COG database and classified into 25 COG classifications with terms abbreviation from A to Z. Among these terms the term R (general function prediction only) gathered the most number of unigenes, followed by L (replication, recombination, and repair) (Fig. 3A). Furthermore, 18,094 unigenes were annotated in the KOG database and clustered into 25 KOG categories with “general function prediction only” (abbreviated as R) containing the greatest number of unigenes, followed by “signal transduction mechanism” (abbreviated as T) (Fig. 3B). Additionally, 8,139 unigenes were annotated in the KEGG database and assigned to 225 KEGG pathways with “Ubiquitin mediated proteolysis” owning the most annotated unigenes (Table S4).

### *Sox* gene identification and phylogenetic analysis

**Figure 4:**
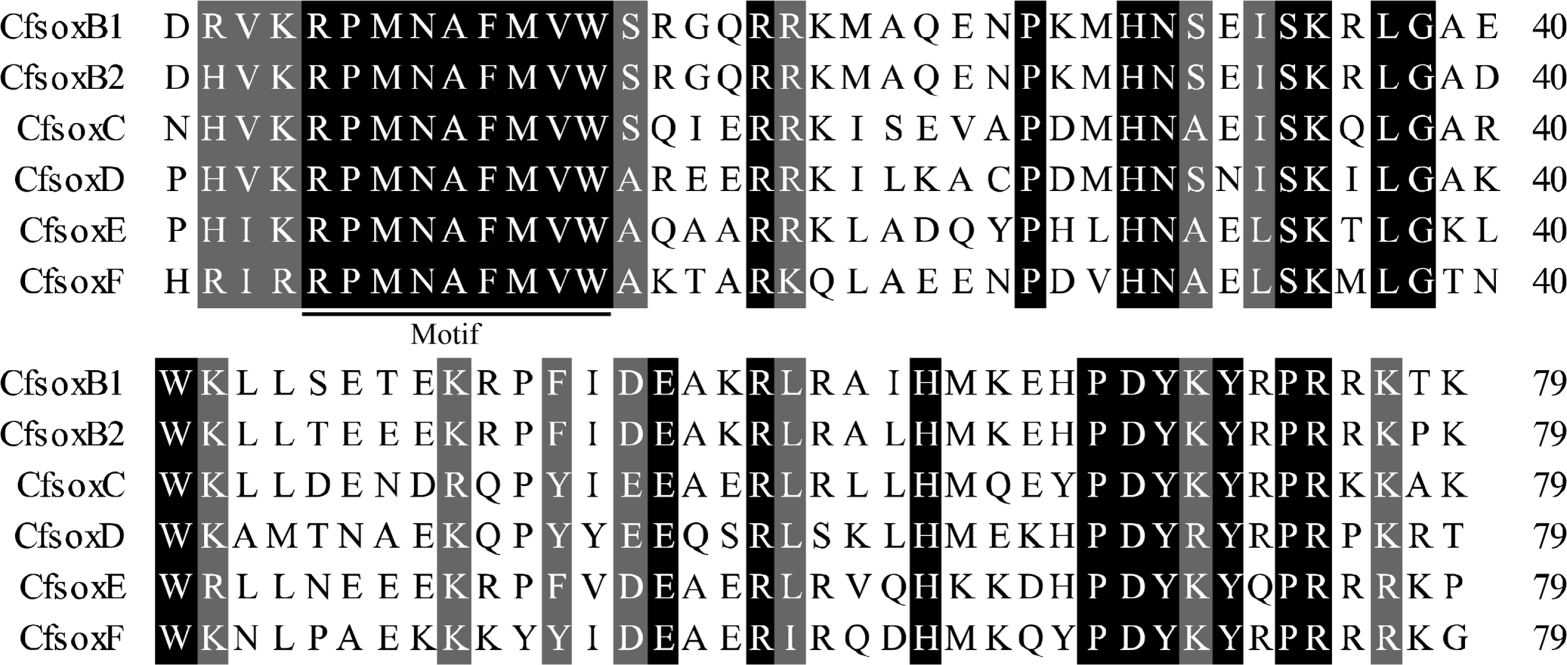
Multiple alignments of HMG-box sequences of SOX in *Corbicula fluminea*. The residues shown with black or gray shadows stand for different extents of conservation. Underlined sequence indicates the conserved motif of HMG. The number at the end of each sequence stands for the amino acid length of HMG.

After local BLAST search throughout the transcriptome of female *C. fluminea*, six *Sox* genes namely *SoxB1*, *SoxB2*, *SoxC*, *SoxD*, *SoxE*, and *SoxF* (GenBank accession number range MH184524 –MH184529) were finally identified, all of which contained a single HMG domain of 79 amino acid residues. Sequence alignment indicated that HMG domains of the six SOX were relatively conserved, and the symbolic motif RPMNAFMVW of SOX family (from five to 13 in amino acid position of HMG) was identical among the six SOX (Fig. 4), indicating the functional importance of the motif. In fact, it is the core domain for recognizing and binding cis-regulatory elements in the promoter region of their target genes (Wei et al., 2016).

**Figure 5:**
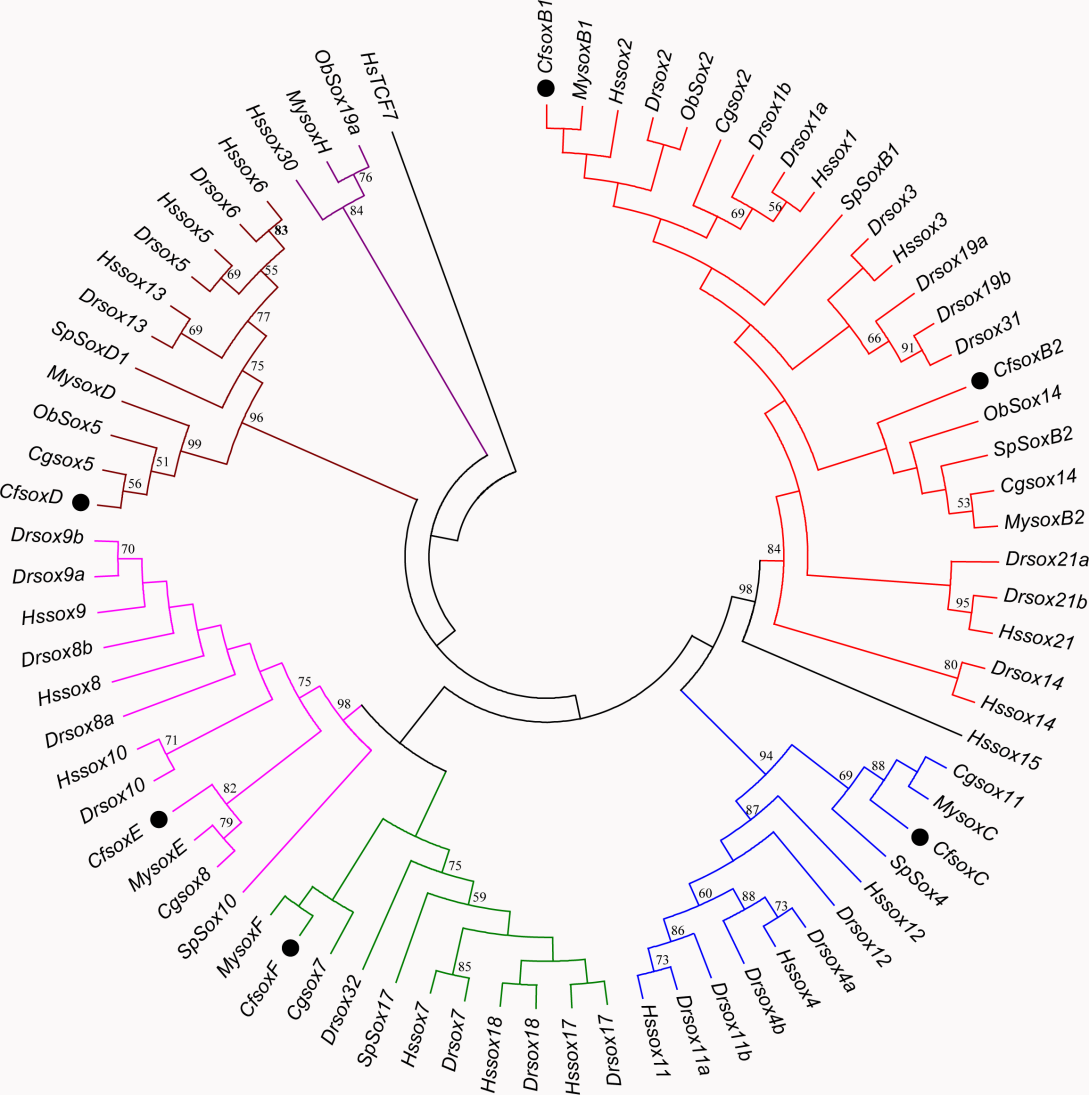
Minimum-Evolution (ME) phylogenetic tree of *Corbicula fluminea* and other species based on HMG domain of SOX proteins. The SOX proteins of *C. fluminea* are marked with black dots. Different Sox groups are denoted with different branch colors: group B (red), C (blue), D (brown), E (magenta), F (green), G (black), and H (purple). Hs,* Homo sapiens*, Dr, *Danio rerio*,** My, *Mizuhopecten yessoensis*, Cf, *Corbicula fluminea*, Cg, *Crassostrea gigas*, Ob,* Octopus bimaculoides*, and Sp,* Strongylocentrotus purpuratus*.

**Figure 6:**
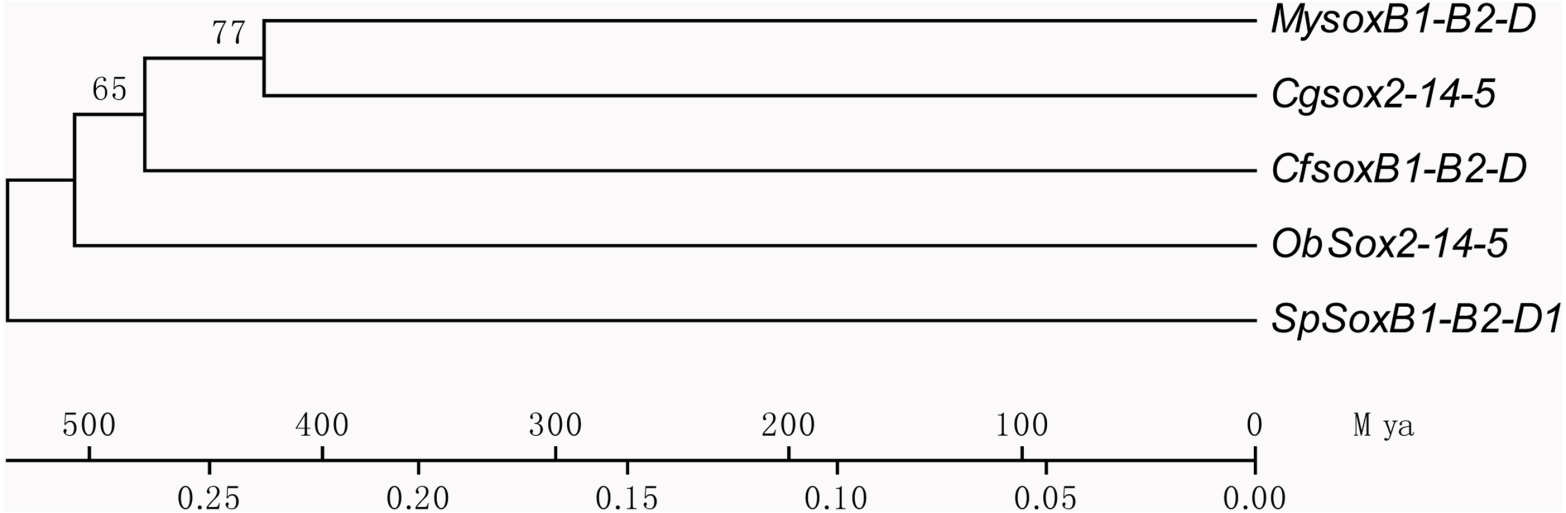
Phylogenetic tree of concatenated dataset of *SoxB1*,* SoxB2*, and *SoxD* using third-codon position substitution rates among *Corbicula fluminea* (Cf) and other species. My, *Mizuhopecten yessoensis*, Cg, *Crassostrea gigas*, Ob,* Octopus bimaculoides*, Sp, *Strongylocentrotus purpuratus*, and Mya, million years ago.

Based on the multiple alignment results of SOX HMG sequences (Fig. S1), the phylogenetic tree was constructed among *C. fluminea*, human, zebrafish, Yesso scallop, oyster, octopus, and sea urchin. The tree formed seven groups which were B, C, D, E, F, G, and H (Fig. 5), and the six SOX obtained in the transcriptome of *C. fluminea* were clustered in five of them (B, C, D, E, and F). Additionally, the UPGMA tree concatenated to date the divergence time between *C. fluminea* and other mollusks showed that the relationship between the two marine bivalves, *M. yessoensis* and *C. gigas*, was the closest. According to the divergence time between scallop (*M. yessoensis*) and oyster (*C. gigas*), which has been reported to be around ∼425 Mya (million years ago) (Wang et al., 2017a), the divergence time between *C. fluminea* and the lineage leading to *M. yessoensis* and *C. gigas* was estimated to be around ∼476 Mya. Furthermore, the bivalve lineage was estimated to be separated with cephalopods around ∼506 Mya (Fig. 6).

### Microsatellite identification and validation

In order to obtain microsatellites containing flanking sequences with enough lengths for primer design, only those unigenes with a length of more than 1 kb were used for SSR detection. Using the software MISA, 20,846 unigenes (total length of 40,396,659 bp) were screened out for microsatellite identification, and SSRs were finally detected in 2673 of them. A total of 3117 SSRs were detected (Table S5), and according to the total length of the 20,846 unigenes, the average distribution density of SSRs was calculated to be 1:12,960 bp with an average SSR frequency of 0.15 (3117/20,846) throughout the transcriptome of *C. fluminea*. Of the 2673 unigenes containing SSRs, 368 (13.8%) owned more than one SSRs. Excluding those containing mononucleotide SSRs, annotation for 1194 SSR-containing unigenes were conducted, 987 of which were successfully annotated. Among the 1194 SSR-containing unigenes, 355 (29.7%) were annotated in GO terms with 137 (38.6%) located in the category “biological process”, 63 (17.7%) in “cellular component” and 155 (43.7%) in “molecular function” (Table S5). Furthermore, 324 of the 1194 SSR-containing unigenes were annotated in the KEGG database and assigned to 60 pathways, among which pathways ko03008 (Ribosome biogenesis in eukaryotes) and ko04120 (Ubiquitin mediated proteolysis) consisted the most SSR-containing unigenes (Table S5). Moreover, 528 (44.2%) of the SSRs were distributed in coding regions (CDS) and the remaining 666 (55.8%) were in untranslated regions (UTR) (Table S5).

Among the identified 3117 SSRs, mono- to penta- nucleotide repeats were detected with mono-nucleotide motifs being the most abundant (1896, 60.8%), followed by tri-nucleotide (887, 28.5%) (Table 3). A total of 35 types of repeat motifs were found in the *C. fluminea* transcriptome. The most abundant motif was A/T (1804, 57.9%), followed by AAC/GTT (352, 11.3%) and AAC/GTT (352, 11.3%) (Table 3, Table S6). For SSRs with di-, tetra-, and penta-nucleotide motifs, the most abundant types were AT/TA (123, 3.9%), ACGG/CCGT (12, 0.4%) and AACAG/CTGTT (7, 0.2%), respectively (Table 3, Table S6).

**Table 3:** Summary of SSRs identified from the transcriptome of *Corbicula fluminea*.

Type	Number	Percentage	Dominant motif	Number of dominant motif	Percentage of dominant motif
Mono-nucleotide	1,896	60.8%	A/T	1,804	57.9%
Di-nucleotide	268	8.6%	AT/TA	123	3.9%
Tri-nucleotide	887	28.5%	AAC/GTT	352	11.3%
Tetra-nucleotide	54	1.7%	ACTC/AGTG	12	0.4%
Penta-nucleotide	12	0.4%	AACAG/CTGTT	7	0.2%
Hexa-nucleotide	0	0.0%	–	0	0.0%
Total	3,117	100%	–	2,298	73.7%

Repeat times of these SSR motifs ranged from 5 to 69. Most SSR motifs repeated 10 times with a percentage of 37.6% (1,172), and the repeat times of 5 (578, 18.5%) and 11 (347, 11.1%) were also common (Table 4). Excluding the mononucleotide types, the copy numbers for most SSRs were from 5 to 10 (1159, 94.9%), and only a small percentage were more than 10 repeat times (62, 5.1%) (Table 4). Finally, 7341 primer pairs (three pairs for each SSR) were designed for 2,447 SSR-containing unigenes which have enough flanking sequence lengths (Table S7).

**Table 4:** Summary of repeat times for different SSRs isolated from the transcriptome of *Corbicula fluminea*.

	5 repeats	6 repeats	7 repeats	8 repeats	9 repeats	10 repeats	11 repeats	12 repeats	13 repeats	14 repeats	15 repeats	>15 repeats	Total
Mono-nucleotide	0	0	0	0	0	1,152	335	137	56	21	16	179	1,896
Di-nucleotide	0	127	51	30	12	9	6	7	1	2	3	20	268
Tri-nucleotide	544	174	82	49	9	9	5	0	2	2	3	8	887
Tetra-nucleotide	27	16	3	4	1	1	0	0	1	1	0	0	54
Penta-nucleotide	7	0	3	0	0	1	1	0	0	0	0	0	12
Total	578	317	139	83	22	1,172	347	144	60	26	22	207	3,117
Percentage	18.5%	10.2%	4.5%	2.7%	0.7%	37.6%	11.1%	4.6%	1.9%	0.8%	0.7%	6.6%	100%

The results of validation indicated that 45 of the 50 microsatellites could be successfully amplified, 31 of which are polymorphic (Table 5). The results of polymorphic characterization for the 31 SSRs in the test population revealed that *Na* ranged from 2 to 9 with an average of 5.5; *He* varied from 0.106 to 0.878 (0.644 on average) and *Ho* ranged from 0.000 to 0.722 (0.380 on average) (Table 5). *PIC* values of the 31 loci varied from 0.099 to 0.843, 23 of which were highly informative (*PIC* >0.5) (Botstein et al., 1980) (Table 5).

**Table 5:** Polymorphic characterization of 31 validated microsatellites developed in transcriptome of *Corbicula fluminea*.

Locus	GenBank accession no.	Repeat Motif	Primer Sequences (5′–3′)	Size (bp)	*Ta* (°C)	*Na*	*He*	*Ho*	*PIC*
cfE02	MF044426	(ATG)19	CTATGAGGAAATCCATTCAC	202–259	54	8	0.805	0.618	0.763
			ATCCCCTTTGTTAGCAGTT						
cfE03	MF044427	(ACA)13	TCACTACTCCGTTGATGTCG	624–630	58	2	0.106	0.000	0.099
			TGCCCGTTGTCATTATCTAT						
cfE04	MF044428	(CAG)10	TCAACGAACAGTACCAGAAG	110–131	52	3	0.133	0.139	0.127
			TACCTGCTCCACTCCAAT						
cfE06	MF044429	(TCA)11	CCTTGTTCACATCGTCACC	135–156	52	4	0.594	0.588	0.526
			CGCAAACACCAAATGTAGAG						
cfE07	MF044430	(GCT)11	CTTTAGCCGCAGATTCCT	191–221	54	7	0.808	0.500	0.770
			CAACGATTTCTTCTTGCCT						
cfE08	MF044431	(CAG)10	TGTTATTCCTATTGTTGGTCC	400–412	54	4	0.727	0.500	0.665
			GATGTTCATTCGCCGTTT						
cfE09	MF044432	(ACA)10	TCGGTCAGCCAATCAAAAC	129–147	52	5	0.727	0.722	0.671
			TGCCATTATCGCTTCAGAGA						
cfE13	MF044433	(TCCG)11	TGGTGTTTATGAACTGTCTGT	122–162	52	5	0.513	0.167	0.476
			ATGCCAATGCTCTTTGTAG						
cfE17	MF044434	(GCAC)6	TGATTTTCACACACATACACG	119–171	52	9	0.871	0.528	0.843
			GTCAGAATAGTCGCACAAGC						
cfE20	MF044435	(ATG)18	ACATCACAGGGACCACTCT	661–706	52	6	0.581	0.139	0.522
			CTCTATCACATATTGCTTTGC						
cfE22	MF044436	(AAC)11	AATGACTGTGTTTATGTGGAC	100–124	52	7	0.485	0.250	0.458
			CAGCATCAGTTTATCACTTG						
cfE25	MF044437	(GCT)11	CAACTGGAAACTTTACGACAT	146–179	52	7	0.835	0.472	0.801
			GGGAAGGAGAAGTAGTAGTGG						
cfE28	MF044438	(ACA)10	AAACTCCCGATACATACAGG	220–235	50	6	0.635	0.571	0.577
			AGATTGTGTCTGAAGTTGAGG						
cfE29	MF044439	(GAA)10	GTTCTAAAAGCGGTTACTGAG	712–724	52	5	0.676	0.400	0.604
			CCATTGGCTGAAAACTGAT						
cfE30	MF044440	(CAG)8	CAACATAATACCCTCCAATCC	388–421	52	6	0.711	0.412	0.658
			TGTGCTTAGTAAAACTCGGC						
cfE31	MF044441	(GAT)8	AGTAGTTACAGCAGTAGCAGC	233–239	52	3	0.621	0.343	0.530
			TCCTGGACTTTCTGATTGAT						
cfE32	MF044442	(TCA)8	GCAGGACTCAACCAGGATT	273–321	52	6	0.709	0.528	0.649
			GAAGCAACCAGTAAAGACAGC						
cfE33	MF044443	(GTG)8	ATCTATGCCCAACAGAACTG	642–702	52	8	0.781	0.294	0.736
			TTTGTAGTCAGGGTTTGAGC						
cfE34	MF044444	(TGC)8	GCATCAAGAAGGCGAAGG	247–280	52	6	0.409	0.306	0.388
			AGCAATGTGTTTTCCAGCA						
cfE35	MF044445	(GTT)8	CACGCTGTAGTCAATCCG	190–202	52	5	0.743	0.500	0.690
			AAGTGTTTGGCTGGTAAGG						
cfE37	MF044446	(AAC)8	ATGTTGTACCTACACCACCT	131–149	52	2	0.460	0.306	0.351
			CGCTAAATGTTCACTACCC						
cfE39	MF044447	(AAC)8	CTGATGACGGACAGTGGAT	680–722	54	7	0.755	0.457	0.718
			AACAAACACGACGGGACT						
cfE40	MF044448	(GCA)8	TGTTGAGAAGAAGCGAGGAT	244–259	54	4	0.720	0.514	0.658
			CTACTGTGGTGTTCAGAATGGT						
cfE41	MF044449	(CAT)8	AACTTTATTATCTGCGTCTTC	122–146	52	6	0.739	0.313	0.683
			AAAATGACCCTCACGATAG						
cfE42	MF044450	(TGA)8	CAGAAGATAGTAGTGGCAGTG	136–166	50	5	0.765	0.471	0.714
			CTGTTGCTCATAACCTCTAAG						
cfE44	MF044451	(ATC)8	GTCTTTCTGGGGCATCACT	613–640	54	6	0.798	0.333	0.758
			TCTTCCAAACGAGGACATTC						
cfE45	MF044452	(ATG)8	GGTAAAGTTTCTACAAGGGAG	149–164	54	6	0.773	0.500	0.725
			GCTGGGTTTAACTGGTCTT						
cfE46	MF044453	(AGC)8	ATGCTGCTCAACTCAATGTG	262–320	56	6	0.603	0.086	0.550
			GTTTTGTGTAGATGTTCTGGC						
cfE47	MF044454	(TCA)8	CTGCTGTCACTGCCTTCAT	177–198	56	5	0.545	0.457	0.495
			GACAAAGAAGCCGCTGATA						
cfE48	MF044455	(TCC)7	AATAGTTCCGTTCTTTGGC	529–550	52	7	0.767	0.333	0.720
			AGATGACCCTGATGCTGATA						
cfE50	MF044456	(TGA)7	AGCCAATCACAGAAAGCC	241–250	56	4	0.558	0.028	0.475
			GTTGAAGCACCCTGACTAAG						
Average	–	–	–	–	–	5.5	0.644	0.380	0.594

## Discussion

### Transcriptome assembly

In this study, all the tissues of *C. fluminea* were used for library construction and RNA-seq to obtain as many expressed sequences as possible. After the assembly of the clean data, 89,563 unigenes were finally obtained, with average and N50 lengths of 859 bp and 1072 bp, respectively. A previous study reported a transcriptome of *C. fluminea* in which 134,684 unigenes were assembled with an average unigene length of 791 bp and 74.4% of the sequences were longer than 500 bp (Chen et al., 2013). Comparatively, both the average length (859 bp) and >500 bp percentage (82.1%) were improved in this present study. In addition, these two data were also higher than those of other mollusks sequenced using the Illumina method, including that of *Cristaria plicata* (737 bp, 34.0%) (Patnaik et al., 2016), *P. textile* (618 bp, 34.8%) (Chen et al., 2016), and *Mizuhopecten yessoensis* (436 bp, 15.1%) (Meng et al., 2013).

### Gene function annotations

Seven databases were used for functional annotation of unigenes, while only a small part (36.7%) of the 89,563 unigenes were successfully annotated, which was similar to that in the previous study on *C. fluminea* transcriptome (Chen et al., 2013). This annotation rate was still higher than those in many previously reported mollusks, such as 21.19% in *Chlamys nobilis* (Liu et al., 2015), 9.9% in *Sinonovacula constricta* (Niu et al., 2013), and 27.78% in *Pinctada maxima* (Deng et al., 2014). In addition, this rate was similar to those of some other bivalve species including *P. textile* (38.92%) (Chen et al., 2016), *P. martensii* (36.19%) (Zhao et al., 2012), and *Pecten maximus* (31%) (Pauletto et al., 2014). Compared to bony fishes such as *Sarcocheilichthys sinensis* (96.2%) (Zhu et al., 2017), *Gymnocypris przewalskii* (73.3%) (Tong et al., 2015), and *Hypophthalmichthys molitrix* (63.2%) (Fu & He, 2012), the annotation rates of unigenes in mollusks seem to be at a much lower level. Although un-annotated unigenes of *C. fluminea* were further searched for ncRNA, only quite a small part of them had homologies. Compared to well-studied model species such as zebrafish, available genomic information of mollusks is insufficient in public databases which may be the most probable reason for the low annotation rate of *C. fluminea* and other mollusks unigenes. Additionally, there was still a probability that un-annotated unigenes may represent novel, fast-evolving, or species-specific genes (Chen et al., 2016) which would provide important information for further research on the function and evolution analysis of genes.

Similarity analysis in the nr database indicated that *C. fluminea* had the most homologous sequences with another bivalve *C. gigas*, which has sufficient sequences in this public database. A total of 10,783 *C. fluminea* unigenes were classified into 55 GO terms, the composition and distribution of which were similar to those of many mollusks, such as 62 GO terms in *P. textile* (Chen et al., 2016), 53 in *S. constricta* (Niu et al., 2013), and 59 in *P. maxima* (Deng et al., 2014). In addition, 7654 (8.5%) unigenes were annotated and classified into 25 COG classifications, and 8139 (9.1%) unigenes were annotated in the KEGG database and assigned to 225 KEGG pathways, both of which were also similar to those in previously reported studies (Niu et al., 2013; Pauletto et al., 2014; Liu et al., 2015; Chen et al., 2016).

### Characterization and phylogenetic analysis of *C. fluminea Sox* genes

Although the number of *Sox* genes varies among different animals, all of them have been classified into 11 *Sox* groups (A-K). Among these groups, B, C, E and F exist in almost all animal lineages, and they are believed to be the core groups (Fortunato et al., 2012; Heenan, Zondag & Wilson, 2016). Previously, *SoxD* was thought to be specific in vertebrates, however, it has already been identified in invertebrates (Bowles, Schepers & Koopman, 2000) such as *Drosophila melanogaster*, *Lingula anatine*, and *M. yessoensis* (Yu et al., 2017). Hence, *SoxD* is also currently accepted as a core group. Others are usually lineage-specific and are called noncore groups, for example, *SoxA* is specific in mammals, *SoxG* in vertebrates, *SoxI* and *SoxJ* in *Caenorhabitis elegans*, and *SoxK* in teleosts (Bowles, Schepers & Koopman, 2000; Wei et al., 2016). In this study, six *Sox* genes (*SoxB1*, *SoxB2*, *SoxC*, *SoxD*, *SoxE*, and *SoxF*) were isolated from the transcriptome of *C. fluminea*, and all of them belong to the core *Sox* groups. Although *SoxH* has been reported in the marine bivalves *M. yessoensis* (Yu et al., 2017) and *C. gigas* (Zhang, Xu & Guo, 2014b), it was not found in *C. fluminea* in this study. The most probable reason for the absence of *SoxH* is that the *C. fluminea* samples used for transcriptome sequencing were females, and *SoxH* is specifically expressed in the testes of *M. yessoensis* and *C. gigas* (Zhang, Xu & Guo, 2014b; Yu et al., 2017). Therefore, this gene cannot be detected in female transcriptome. Another possibility is that *SoxH* may have been lost during genome duplication and remodeling in *C. fluminea*, which has been observed in other animals (Heenan, Zondag & Wilson, 2016). Thus, further studies on male transcriptome or whole genome are needed to investigate the occurrence of *SoxH* in *C. fluminea*.

Similar to previous studies, *SoxB1* and *SoxB2* groups could not be clearly separated in the phylogenetic tree of this study, which was constructed using HMG box protein sequences, due to their high sequence similarity of HMG domains (Fortunato et al., 2012; Heenan, Zondag & Wilson, 2016). Through the phylogenetic tree, it was easy to observe that most *Sox* genes of bivalves (*C. fluminea*, *M. yessoensis*, and *C. gigas*) were not clustered with those of vertebrates (human and zebrafish). Instead, they formed separate sub-branches, indicating that the number of *Sox* genes increased after the separation of vertebrates. It has been reported that two whole genome duplication (WGD) events have occurred around 520–550 Mya in the vertebrate lineage (Blomme et al., 2006), and members of the *Sox* gene families were believed to increase following WGD though duplication and loss of their ancestral genes in different vertebrate phyla (Meyer & Van de Peer, 2005).

Using *Sox* genes as a molecular clock, times of origin have been estimated in many aquatic animals, especially in teleosts (Zhong, Yu & Tong, 2006; Guo, Tong & He, 2009; Guo, Yu & Tong, 2014), however, such reports are limited in mollusks. Following reported approaches in these studies, the time of origin of clam was dated back to around ∼476 Mya according to the divergence time between scallop and oyster (Wang et al., 2017a). Meanwhile, the bivalve lineage was estimated to be separated with cephalopods around ∼506 Mya indicating that the appearance of bivalves may be around this period, which was quite similar to that estimated through scallop genome sequences (∼504 Mya) (Wang et al., 2017a). These results would provide valuable reference for evolutionary analysis of *C. fluminea* and bivalves.

### SSR characterization in transcriptome of *C. fluminea*

Out of the 20,846 unigenes that are longer than 1 kb, 3117 SSRs were detected from 2673 (12.8%) of them with an average SSR distribution density of 1:12,960 bp. The average SSR distribution density was 0.15 SSR per unigene, which was similar to that of *C. virginica* (0.15) (Zhang et al., 2014a) and *P. textile* (0.10) (Chen et al., 2016), and higher than that of *C. nobilis* (0.03) (Liu et al., 2015), *C. plicata* (0.05) (Patnaik et al., 2016), and *S. constricta* (0.09) (Niu et al., 2013). The percentage of unigenes that possess potential SSRs in this study (12.8%) was similar to that of *P. textile* (10%) (Chen et al., 2016), *Hyriopsis cumingii* (8.3%) (Bai et al., 2013), and *C. plicata* (16.3%) (Patnaik et al., 2016). The distribution density of SSRs throughout *C. fluminea* transcriptome was higher than that in *P. maxima* (Deng et al., 2014), but lower than that in *C. plicata* (Patnaik et al., 2016) and *C. nobilis* (Liu et al., 2015). The variety of SSR distribution densities among organisms may be due to several probable reasons such as differences in genome structures and compositions (Toth, Gaspari & Jurka, 2000), varied sizes of transcriptome dataset, different parameters and criteria used for SSR detection (Varshney, Graner & Sorrells, 2005).

Out of the identified 3117 SSRs, mono-nucleotide repeat was the most abundant, as reported in other aquatic animals (Zhang et al., 2014a; Li et al., 2015b; Zhu et al., 2017). However, mononucleotide repeat SSRs were usually excluded for characterization and even not considered during SSR detection (Li et al., 2015b; Tong et al., 2015; Chen et al., 2016), because of their lower application value caused by potential inaccurate sequence information (Li et al., 2015b). If mononucleotide repeats were excluded, the most abundant SSR motif became tri-nucleotide repeats (72.6%) in the *C. fluminea* transcriptome. A previous study on *C. fluminea* also reported that tri-nucleotide repeat SSR was the dominant type with a rate of 57.8% (Chen et al., 2013). Similarly, in *P. textile* (53.0%) (Chen et al., 2016) and *S. constricta* (46.4%) (Niu et al., 2013) tri-nucleotide repeat SSR was also the dominant type. However, in other bivalves such as *P. maxima* (79.4%) (Deng et al., 2014), *H. cumingii* (46.9%) (Bai et al., 2013), *C. virginica* (63.4%) (Zhang et al., 2014a) and *C. plicata* (65.5%) (Patnaik et al., 2016), the dominate type was di-nucleotide repeat SSRs. These results indicate that the genome composition of bivalves from different taxonomic groups may be quite different. It has been reported that the most abundant repeat motif in vertebrate is AC/GT (Brenner et al., 1993), however, the richest motif may be different in mollusks. For example, in *C. fluminea* (this study), *S. constricta* (Niu et al., 2013), *C. nobilis* (Liu et al., 2015), and *Mytilus* spp. (Malachowicz & Wenne, 2019), the dominate motif for di-nucleotide repeat SSRs was AT/TA, which would provide useful information for further studies on evolution of SSRs.

### SSR validation and polymorphism analysis

Among the randomly selected 50 SSR primer pairs, 45 (90%) could be successfully amplified, 31 (68.9%) of which were polymorphic. Comparatively, the success rate (90%) in this study was much higher than those in previous studies, for example, 53.8% success rate was observed in *P. textile* (Chen et al., 2016), 63.8% in *P. maxima* (Deng et al., 2014), and 65.5% in *S. constricta* (Niu et al., 2013). The higher success rate may be the result of our manual adjustments for some of the selected primer pairs which had too low GC rates, formed dimers, or more than three repeated nucleotides at the 3′ends. These results also indicate that most SSRs predicted in the transcriptome of *C. fluminea* were reliable.

Of the validated 45 SSRs, 68.9% were polymorphic, which was similar to the percentages observed in *P. maxima* (66.7%) (Deng et al., 2014) and in *S. constricta* (72.2%) (Niu et al., 2013), but was lower than that observed in *P. textile* (83.7%) (Chen et al., 2016). In spite of this, 74.2% of the polymorphic SSRs were highly informative (*PIC* > 0.5), indicating their potential usage in future studies. In summary, the expressed sequence tags (EST) related SSRs identified in transcriptome of *C. fluminea* would be useful for further genetic and genomic studies including population structure analyses, genetic linkage map construction, comparative genome mapping, QTL identification, and MAS breeding in this species.

A transcriptome of *C. fluminea* was reported in a previous study (Chen et al., 2013), however, the study had many limitations such as unknown sex of samples, relatively higher error rate of reads and assembly, insufficent analysis of SSRs. In addition, the search for un-annotated unigenes was not carried out in the databases of non-coding RNAs. The quality of transcriptome information reported in this study is an improvement on that from the previous study. Firstly, the whole soft tissues were used for library construction and RNA-seq, which allows the collection of gene sequences not expressed in the five tissues (mantle, muscle, digestive gland, gonad and gill) analyzed in the previous study. Secondly, the sex of *C. fluminea* samples was clear, which made it easy to extract interested gene information in female *C. fluminea* for scholars who are interested in sex determination. Thirdly, the sequencing platform used in this study (Illumina HiSeq 2500) could produce a longer read length of 125 bp, and the standard for high-quality reads was Q30 (the error rate of nucleotide was 0.1%), both of which could confirm the accuracy of assembled unigenes. Fourthly, un-annotated unigenes were searched in databases of non-coding RNAs, and we clarified that the reason for the low annotation rate of unigenes was not from non-coding RNAs. Finally, we made deeper analyses on SSRs: microsatellite were identified only in unigenes with a length of more than 1,000 bp; three pairs of primers were designed for each SSR loci; SSR-containing sequences were annotated; positions of SSRs (in CDS or UTR) were clarified; and 50 SSRs were validated and characterized in a test population.

## Conclusions

Using the high-throughput Illumina HiSeq 2500 platform, the transcriptome of whole soft tissues was assembled, characterized, and annotated in Asian clam. Six *Sox* genes were identified and a set of SSRs were also isolated. These data gave us an overview of the transcriptome of adult female *C. fluminea*, and will provide useful information for further studies on genes of interest. The *Sox* genes will be helpful for origin and evolution analyses of clams and bivalves. Furthermore, thousands of isolated EST-SSRs would be useful tools for future genetic and genomic studies in *C. fluminea* and its closely related species.

##  Supplemental Information

10.7717/peerj.7770/supp-1 Table S1 *Sox* genes used for phylogenetic study in this study

10.7717/peerj.7770/supp-2 Table S2 Information of integrated function annotation of *Corbicula fluminea* unigenes

10.7717/peerj.7770/supp-3 Table S3 The results of homologous analysis against the noncoding RNA database for un-annotated *Corbicula fluminea* unigenes

10.7717/peerj.7770/supp-4 Table S4 KEGG classification of *Corbicula fluminea* unigenes

10.7717/peerj.7770/supp-5 Table S5 Basic information of SSRs and annotation of SSR-containing sequences in the transcriptome of *Corbicula fluminea* a: Mononucleotide repeat SSRs were excluded; p2-p5 stand for di- to penta-nucleotide motifs respectively; c stand for compound types. *: Indicating SSR sequences that Primers couldn’t be designed. N/A: No annotations in any of the 9 databases. –: No annotations in a given database.

10.7717/peerj.7770/supp-6 Table S6 Frequency of different SSR types in the transcriptome of *Corbicula fluminea*

10.7717/peerj.7770/supp-7 Table S7 Information of primers designed for SSRs developed from the transcriptome of *Corbicula fluminea*

10.7717/peerj.7770/supp-8 Figure S1 Multiple alignment of HMG-box domain sequences of SOX proteins from *Corbicula fluminea* and other species Hs,* Homo sapiens*, Dr, *Danio rerio*,** My, *Mizuhopecten yessoensis*, Cf, *Corbicula fluminea*, Cg, *Crassostrea gigas*, Ob,* Octopus bimaculoides*, and Sp,* Strongylocentrotus purpuratus*.

10.7717/peerj.7770/supp-9 Supplemental Information 1 Raw data of polymorphic microsatellite characterization

10.7717/peerj.7770/supp-10 Supplemental Information 2 Sequences of the 89,563 assembled unigenes of the *Corbicula fluminea* transcriptome

## Additional Information and Declarations

### DNA Deposition

The following information was supplied regarding the deposition of DNA sequences: Sequences are available at NCBI: MH184524–MH184529 and MF044426–MF044456.

### Data Availability

The following information was supplied regarding data availability: Raw data is available at SRA under accessions SRX2786025 and SRR5512046.
