# Supplementary material for: Transcriptome-wide identification and characterization of the Sox gene family and microsatellites for Corbicula fluminea
Source: PeerJ. 2019 Oct 22;7:e7770. doi: 10.7717/peerj.7770 (PMC6814067; doi:10.7717/peerj.7770)
Supplement: Figure S1 — Hs, Homo sapiens, Dr, Danio rerio, My, Mizuhopecten yessoensis, Cf, Corbicula fluminea, Cg, Crassostrea gigas, Ob, Octopus bimaculoides, and Sp, Strongylocentrotus purpuratus. [file peerj-07-7770-s008.pdf]

MysoxH	GKVKRPMNAFMVWARNYRSRLSEEMPQASNAQISVRLGQIWGSMPLADKEKYFREAERIKLQHNRDFPGWVYQPKQ
ObSox19a	GNIKRPMNAFMVFGRTYRSTLMKMFPTASNSQISIYLGEIWHKMSPAQQKPYFDESNRIKYKHRKDYPGWVYRPNI
Hssox30	GHVKRPMNAFMVWARIHRPALAKANPAANNAEISVQLGLEWNKLSEEQKKPYYDEAQKIKEKHREEFPGWVYQPRP
Drsox5	PHIKRPVNAFMVWAKDERRKILQAFPDMHNSNISKILGSRWKSMTNLEKQPYYEEQARLSKQHLEKYPDYKYKPRP
Hssox5	PHIKRPMNAFMVWAKDERRKILQAFPDMHNSNISKILGSRWKAMTNLEKQPYYEEQARLSKQHLEKYPDYKYKPRP
Drsox6	PHIKRPMNAFMVWAKDERRKILQAFPDMHNSNISKILGSRWKSMTNQEKQPYYEEQARLSKIHLEKYPNYKYKPRP
Hssox6	PHIKRPMNAFMVWAKDERRKILQAFPDMHNSNISKILGSRWKSMSNQEKQPYYEEQARLSKIHLEKYPNYKYKPRP
Drsox13	GHIKRPMNAFMVWAKDERRRILQAFPDMHNSSISKILGSRWKSMSNQEKQPYYEEQARLSRQHLERYPDYKYKPRP
Hssox13	SHIKRPMNAFMVWAKDERRKILQAFPDMHNSSISKILGSRWKSMTNQEKQPYYEEQARLSRQHLEKYPDYKYKPRP
SpSoxD1	PHIKRPMNAFMVWAKEERRKILARHPDMHNSNISKILGSKWKTMSNAEKQPYYEEQARLSKAHLEKYPDYKYKPRP
CfsoxD	PHVKRPMNAFMVWAREERRKILKACPDMHNSNISKILGAKWKAMTNAEKQPYYEEQSRLSKLHMEKHPDYRYRPRP
Cgsox5	PHIKRPMNAFMVWAREERRKILKACPDMHNSNISKILGAKWKAMTNAEKQPYYEEQSRLSKLHMEKHPDYRYRPRP
ObSox5	PHVKRPMNAFMVWAREERRKILKACPDMHNSNISKILGAKWKAMSNAEKQPYYEEQSRLSKLHMEKHPDYRYRPRP
MysoxD	PHIKRPMNAFMVWAREERRKILKACPDMHNSNISKILGAKWKAMSNAEKQPFYEEQSRLSKLHMEKHPDYRYRPRP
Drsox4a	GHIKRPMNAFMVWSQIERRKIMEQSPDMHNAEISKRLGKRWKLLKDSDKIPFIREAERLRLKHMADYPDYKYRPRK
Hssox4	GHIKRPMNAFMVWSQIERRKIMEQSPDMHNAEISKRLGKRWKLLKDSDKIPFIREAERLRLKHMADYPDYKYRPRK
Drsox4b	GHIKRPMNAFMVWSQIERRKIMEQSPDMHNAEISKRLGKRWKLLKDGDKIPFIREAERLRLKHMADYPDYKYRPRK
Drsox11a	GHIKRPMNAFMVWSKIERRKIMEQSPDMHNAEISKRLGKRWKMLKDSEKIPFIREAERLRLKHMADYPDYKYRPKK
Hssox11	GHIKRPMNAFMVWSKIERRKIMEQSPDMHNAEISKRLGKRWKMLKDSEKIPFIREAERLRLKHMADYPDYKYRPRK
Drsox11b	GHIKRPMNAFMVWSKIERRKIMEQSPDMHNAEISKRLGKRWKMLKDSEKIPFIREAERLRLQHMADYPDYKYRPKK
Drsox12	GHIKRPMNAFMVWSQIERRKIMEQWPDMHNAEISKRLGKRWKLLPDYEKIPFIKEAERLRLKHMADYPDYKYRPRK
Hssox12	GHIKRPMNAFMVWSQHERRKIMDQWPDMHNAEISKRLGRRWQLLQDSEKIPFVREAERLRLKHMADYPDYKYRPRK
Cgsox11	NHVKRPMNAFMVWSQLERRKISEVSPDMHNAEISKRLGKRWKTLNETDRQPFIEEAERLRLLHMQEYPDYKYRPRK
MysoxC	NHVKRPMNAFMVWSQIERRRISEVSPDMHNAEISKRLGKRWKLLTETERQPYIEEAERLRLLHMQEYPDYKYRPRK
CfsoxC	NHVKRPMNAFMVWSQIERRKISEVAPDMHNAEISKQLGARWKLLDENDRQPYIEEAERLRLLHMQEYPDYKYRPRK
SpSox4	GHIKRPMNAFMVWSQIERRRIMETTPDMHNAEISKRLGRRWKTLDEVAKSPYVEEAERLRLLHMAQYPDYKYRPRK
Drsox3	DRVKRPMNAFMVWSRGQRRKMAQENPKMHNSEISKRLGADWKLLTDAEKRPFIDEAKRLRAMHMKEHPDYKYRPRR
Hssox3	DRVKRPMNAFMVWSRGQRRKMALENPKMHNSEISKRLGADWKLLTDAEKRPFIDEAKRLRAVHMKEYPDYKYRPRR
Drsox19b	DKVKRPMNAFMVWSRGQRRKMAQENPKMHNSEISKRLGAEWKLLTDVEKRPFIDEAKRLRAVHMKEYPDYKYKPRR
Drsox31	DKVKRPMNAFMVWSRGQRRKMAQENPKMHNSEISKRLGAEWKLLTDVEKRPFIDEAKRLRAVHMKEYPDYKYKPRR
Drsox19a	DKVKRPMNAFMVWSRGQRRKMAQENPKMHNSEISKRLGAEWKLLTDAEKRPFIDEAKRLRALHMKEYPDYKYKPRR
CfsoxB2	DHVKRPMNAFMVWSRGQRRKMAQENPKMHNSEISKRLGADWKLLTEEEKRPFIDEAKRLRALHMKEHPDYKYRPRR
Drsox14	DHIKRPMNAFMVWSRGQRRKMAQENPKMHNSEISKRLGAEWKLLSESEKRPYIDEAKRLRAQHMKEHPDYKYRPRR
Hssox14	DHIKRPMNAFMVWSRGQRRKMAQENPKMHNSEISKRLGAEWKLLSEAEKRPYIDEAKRLRAQHMKEHPDYKYRPRR
Drsox2	DRIKRPMNAFMVWSRGQRRKMAQENPKMHNSEISKRLGAEWKLLSESEKRPFIDEAKRLRALHMKEHPDYKYRPRR
Drsox1a	DRVKRPMNAFMVWSRGQRRKMAQENPKMHNSEISKRLGAEWKVMSEAEKRPFIDEAKRLRAMHMKEHPDYKYRPRR

Hssox1	DRVKRPMNAFMVWSRGQRRKMAQENPKMHNSEISKRLGAEWKVMSEAEKRPFIDEAKRLRALHMKEHPDYKYRPRR
Drsox1b	DRVKRPMNAFMVWSRGQRRKMAQENPKMHNSEISKRLGAEWKLMSEAEKRPFIDEAKRLRAMHMKEHPDYKYRPRR
Cgsox2	DRVKRPMNAFMVWSRGQRRKMAQENPKMHNSEISKRLGAEWKLLSEAEKRPFIDEAKRLRAIHMKEHPDYKYRPRR
ObSox2	DRVKRPMNAFMVWSRGQRRKMAQENPKMHNSEISKRLGAEWKLLSESEKRPFIDEAKRLRAIHMKEHPDYKYRPRR
CfsoxB1	DRVKRPMNAFMVWSRGQRRKMAQENPKMHNSEISKRLGAEWKLLSETEKRPFIDEAKRLRAIHMKEHPDYKYRPRR
MysoxB1	DRVKRPMNAFMVWSRGQRRKMAQENPKMHNSEISKRLGAEWKLLTETEKRPFIDEAKRLRAIHMKEHPDYKYRPRR
Hssox2	DRVKRPMNAFMVWSRGQRRKMAQENPKMHNSEISKRLGAEWKLLSETEKRPFIDEAKRLRALHMKEHPDYKYRPRR
SpSoxB1	DRVKRPMNAFMVWSRGQRRKLSQENPKMHNSEISKRLGAEWKLLSEDEKRPFIDEAKRLRAVHMKEHPDYKYRPRR
Cgsox14	EHVKRPMNAFMVWSRGQRRKMAQDNPKMHNSEISKRLGAEWKLLSEEDKRPFIDEAKRLRALHMKEHPDYKYRPRR
MysoxB2	DHVKRPMNAFMVWSRGQRRKMAQENPKMHNSEISKRLGAEWKLLSEEDKRPFIDEAKRLRALHMKEHPEYKYRPRR
SpSoxB2	DHVKRPMNAFMVWSRGQRRKLAQENPKMHNSEISKRLGAEWKLLSEDDKRPFIDEAKRLRALHMKEHPDYKYRPRR
ObSox14	EHVKRPMNAFMVWSRGQRRKMAQENPKMHNSEISKRLGAEWKLLSEEEKRPFIDEAKRLRALHMKEHPDYKYRPRR
Drsox21b	DHVKRPMNAFMVWSRAQRRKMAQENPKMHNSEISKRLGAEWKLLTESEKRPFIDEAKRLRAMHMKEHPDYKYRPRR
Hssox21	DHVKRPMNAFMVWSRAQRRKMAQENPKMHNSEISKRLGAEWKLLTESEKRPFIDEAKRLRAMHMKEHPDYKYRPRR
Drsox21a	DHVKRPMNAFMVWSRAQRRKMALDNPKMHNSEISKRLGGEWKLLSDSEKRPFIDEAKRLRAVHMKEHPDYKYRPRR
Hssox15	EKVKRPMNAFMVWSSAQRRQMAQQNPKMHNSEISKRLGAQWKLLDEDEKRPFVEEAKRLRARHLRDYPDYKYRPRR
Drsox7	PRIRRPMNAFMVWAKDERKRLAVQNPDLHNAELSKMLGKSWKALTPPQKRPYVEEAERLRVQHMQDYPNYKYRPRR
Hssox7	SRIRRPMNAFMVWAKDERKRLAVQNPDLHNAELSKMLGKSWKALTLSQKRPYVDEAERLRLQHMQDYPNYKYRPRR
Drsox17	PRIRRPMNAFMVWAKDERKRLAQQNPDLHNAELSKMLGKSWKALPMVDKRPFVEEAERLRVKHMQDHPNYKYRPRR
Hssox17	SRIRRPMNAFMVWAKDERKRLAQQNPDLHNAELSKMLGKSWKALTLAEKRPFVEEAERLRVQHMQDHPNYKYRPRR
Drsox18	SRIRRPMNAFMVWAKDERKRLAIQNPDLHNAVLSKMLGQSWKALSTLDKRPFVEEAERLRLQHLQDHPNYKYRPRR
Hssox18	SRIRRPMNAFMVWAKDERKRLAQQNPDLHNAVLSKMLGKAWKELNAAEKRPFVEEAERLRVQHLRDHPNYKYRPRR
SpSox17	ARIRRPMNAFMVWAKDERKRLADQNPDLHNADLSKLLGKAWRSLTLMQKQPFVEEAERLRLKHMADHPDYKYRPRR
Drsox32	TRVRRPLNAFIIWTKEERRRLAQLNPDLENTDLSKILGKTWKAMSLADKRPYMQEAERLRIQHTIDYPNYKYRPRR
CfsoxF	HRIRRPMNAFMVWAKTARKQLAEENPDVHNAELSKMLGTNWKNLPAEKKKYYIDEAERIRQDHMKQYPDYKYRPRR
Cgsox7	ERIRRPMNAFMVWAKTERKKLALENPEVHNADLSKILGQKWRELSPSQKKYYSEEADKLRDLHMQKYPDYKYRPRR
MysoxF	QRIRRPMNAFMVWARTERKRLAHENPDVHNADLSKILGKKWKSLPTEEKKIFIDEAERLRVRHMQEHPDYKYKPRR
Drsox8b	PHVKRPMNAFMVWAQAARRKLADQYPHLHNAELSKTLGKLWRLLTESEKRPFVEEAERLRVQHKKDHPDYKYQPRR
Hssox8	PHVKRPMNAFMVWAQAARRKLADQYPHLHNAELSKTLGKLWRLLSESEKRPFVEEAERLRVQHKKDHPDYKYQPRR
Drsox9a	PHVKRPMNAFMVWAQAARRKLADQYPHLHNAELSKTLGKLWRLLNEVEKRPFVEEAERLRVQHKKDHPDYKYQPRR
Drsox9b	PHVKRPMNAFMVWAQAARRKLADQYPHLHNAELSKTLGKLWRLLNEGEKRPFVEEAERLRVQHKKDHPDYKYQPRR
Hssox9	PHVKRPMNAFMVWAQAARRKLADQYPHLHNAELSKTLGKLWRLLNESEKRPFVEEAERLRVQHKKDHPDYKYQPRR
Drsox8a	PHVKRPMNAFMVWAQAARRKLADQYPHLHNAELSKTLGKLWRLLSESEKRPFVEEAERLRLQHKKDYPDYKYQPRR
Cgsox8	PHIKRPMNAFMVWAQAARRKLADQYPHLHNAELSKTLGKLWRLLNEEEKRPFVDEAERLRVQHKKDYPDYKYQPRR
MysoxE	PHIKRPMNAFMVWAQAARRKLADQYPHLHNAELSKTLGKLWRLLNEEEKRPFVDEAERLRVQHKKDYPDYKYQPRR
CfsoxE	PHIKRPMNAFMVWAQAARRKLADQYPHLHNAELSKTLGKLWRLLNEEEKRPFVDEAERLRVQHKKDHPDYKYQPRR
Drsox10	PHVKRPMNAFMVWAQAARRKLADQYPHLHNAELSKTLGKLWRLLNETDKRPFIEEAERLRKQHKKDYPEYKYQPRR
Hssox10	PHVKRPMNAFMVWAQAARRKLADQYPHLHNAELSKTLGKLWRLLNESDKRPFIEEAERLRMQHKKDHPDYKYQPRR
SpSox10	PHIKRPMNAFMVWAQAARKKLGNQYPQLHNAELSKTLGKLWRLLSDKEKQPFIEEAERLRQQHKKDYPDYKYQPRR
